# Intrafamilial variability in *SLC6A1*-related neurodevelopmental disorders

**DOI:** 10.3389/fnins.2023.1219262

**Published:** 2023-07-12

**Authors:** Benedetta Kassabian, Christina Dühring Fenger, Marjolaine Willems, Angel Aledo-Serrano, Tarja Linnankivi, Pamela Pojomovsky McDonnell, Laina Lusk, Birgit Susanne Jepsen, Michael Bayat, Anja A. Kattentidt-Mouravieva, Anna Abulí Vidal, Gabriel Valero-Lopez, Helena Alarcon-Martinez, Kimberly Goodspeed, Marjon van Slegtenhorst, Tahsin Stefan Barakat, Rikke S. Møller, Katrine M. Johannesen, Guido Rubboli

**Affiliations:** ^1^Department of Epilepsy Genetics and Precision Medicine, Danish Epilepsy Center, Member of the European Reference Network EpiCARE, Dianalund, Denmark; ^2^Neurology Unit, Department of Neuroscience, University of Padua, Padua, Italy; ^3^Amplexa Genetics, Odense, Denmark; ^4^Département Génétique Médicale, Maladies Rares et Médecine Personnalisée, Hôpital Arnaud de Villeneuve, CHU de Montpellier Institute for Neurosciences of Montpellier, Univ Montpellier, INSERM, Montpellier, France; ^5^Epilepsy and Neurogenetics Program—Vithas Madrid La Milagrosa University Hospital, Vithas Hospital Group, Madrid, Spain; ^6^Department of Pediatric Neurology, New Children's Hospital and Pediatric Research Center, Epilepsia Helsinki, Helsinki University Hospital and University of Helsinki, Helsinki, Finland; ^7^Division of Neurology, Children's Hospital of Philadelphia, Philadelphia, PA, United States; ^8^Department of Neurology, University of Pennsylvania Perelman School of Medicine, Philadelphia, PA, United States; ^9^Epilepsy Neurogenetics Initiative, Division of Neurology, Children's Hospital of Philadelphia, Philadelphia, PA, United States; ^10^Pediatric Department, Danish Epilepsy Center, Dianalund, Denmark; ^11^Department of Neurology and Center for Rare Diseases, Aarhus University Hospital, Aarhus, Denmark; ^12^Stichting Zuidwester, Middelharnis, Netherlands; ^13^Department of Clinical and Molecular Genetics, University Hospital Vall d’Hebron and Medicine Genetics Group Vall d'Hebron Research Institute (VHIR), Barcelona, Spain; ^14^Neurology Department, Virgen de la Arrixaca University Hospital, Murcia, Spain; ^15^Department of Pediatric Neurology, Virgen de la Arrixaca University Hospital, Murcia, Spain; ^16^Department of Pediatrics, Division of Neurology, University of Texas Southwestern Medical Center, Dallas, TX, United States; ^17^Department of Neurology, University of Texas Southwestern Medical Center, Dallas, TX, United States; ^18^Department of Clinical Genetics, Erasmus MC University Medical Center, Rotterdam, Netherlands; ^19^Discovery Unit, Department of Clinical Genetics, Erasmus MC University Medical Center, Rotterdam, Netherlands; ^20^ENCORE Expertise Center for Neurodevelopmental Disorders, Erasmus MC University Medical Center, Rotterdam, Netherlands; ^21^Institute of Regional Health Research, University of Southern Denmark, Odense, Denmark; ^22^Department of Genetics, University Hospital of Copenhagen, Rigshospitalet, Copenhagen, Denmark; ^23^Department of Clinical Medicine, Faculty of Health and Medical Sciences, University of Copenhagen, Copenhagen, Denmark

**Keywords:** SLC6A1, intrafamilial variability, epilepsy, neurodevelopmental disorders, intellectual disability

## Abstract

**Introduction:**

Phenotypic spectrum of *SLC6A1*-related neurodevelopmental disorders (*SLC6A1*-NDD) includes intellectual disability (ID), autistic spectrum disorders (ASD), epilepsy, developmental delay, beginning from early infancy or after seizure onset, and other neurological features such as hypotonia and movement disorders. Data on familial phenotypic heterogeneity have been rarely reported, thus in our study we aimed to investigate intrafamilial phenotypic variability in families with *SLC6A1* variants.

**Methods:**

We collected clinical, laboratory and genetic data on 39 individuals, including 17 probands, belonging to 13 families harboring inherited variants of *SLC6A1*. Data were collected through an international network of Epilepsy and Genetic Centers.

**Results:**

Main clinical findings in the whole cohort of 39 subjects were: (a) epilepsy, mainly presenting with generalized seizures, reported in 71% of probands and 36% of siblings or first/second-degree relatives. Within a family, the same epilepsy type (generalized or focal) was observed; (b) ID reported in 100% and in 13% of probands and siblings or first/second-degree relatives, respectively; (c) learning disabilities detected in 28% of the *SLC6A1* carriers, all of them were relatives of a proband; (d) around 51% of the whole cohort presented with psychiatric symptoms or behavioral disorders, including 82% of the probands. Out of the 19 patients with psychiatric symptoms, ASD were diagnosed in 40% of them; (e) neurological findings (primarily tremor and speech difficulties) were observed 38.5% of the whole cohort, including 10 probands. Our families harbored 12 different *SLC6A1* variants, one was a frameshift, two stop-gain, while the remaining were missense. No genotype–phenotype associations were identified.

**Discussion:**

Our study showed that first-or second-degree relatives presented with a less severe phenotype, featuring mainly mild intellectual and/or learning disabilities, at variance with the probands who suffered from moderate to severe ID, generalized, sometimes intractable, epileptic seizures, behavioral and psychiatric disorders. These findings may suggest that a proportion of individuals with mild *SLC6A1*-NDD might be missed, in particular those with an older age where genetic testing is not performed. Further studies on intrafamilial phenotypic variability are needed to confirm our results and possibly to expand the phenotypic spectrum of these disorders and benefit genetic counseling.

## Introduction

Inhibitory and excitatory synapses enable interaction between neurons in the brain. GABA (γ-aminobutyric acid) is the main inhibitory transmitter in the mammalian brain, and it is regulated through specialized molecular mechanisms mediating its transport, sequestration, synthesis, and degradation (Conti et al., 1998; Roth and Draguhn, 2012). *SLC6A1* encodes the GABA transporter 1 (GAT-1) that is located on presynaptic neurons and glia, which removes GABA from the extracellular space of the synapse (Razik et al., 2013). In GAT-1 knockout mice, spontaneous spike–wave discharges and absence seizures are observed as the result of an increased tonic inhibition due to an increase of GABA in the extracellular cleft due to defective GAT-1 function (Richards et al., 1995; Belelli et al., 2009; Cope et al., 2009). Similar findings were observed in animal models of generalized epilepsies treated with GABA uptake inhibitors (Coenen et al., 1995), while anti-seizure medications that increase GABA levels, tiagabine, and vigabatrin, worsen or elicit absence seizures (Perucca et al., 1998; Genton, 2000; Bouwman et al., 2007).

Pathogenic variants of *SLC6A1* were first described in exome sequencing studies for intellectual disability (ID) or autism (Rauch et al., 2012; Sanders et al., 2012). In 2014, a patient with myoclonic atonic epilepsy (MAE) and a 3p microdeletion encompassing *SLC6A1* was described (Dikow et al., 2014). This report was followed by a study reporting seven patients with pathogenic *SLC6A1* variants and myoclonic atonic epilepsy (MAE; Carvill et al., 2015). Functional assays on pathogenic *SLC6A1* variants, show a decrease of GABA transport by GAT-1 through defective transport or nonsense-mediated decay, leading to an accumulation of GABA in the intercellular space (Mattison et al., 2018; Cai et al., 2019; Wang et al., 2020; Cheng and Carvill, 2021; Mermer et al., 2021, 2022; Poliquin et al., 2021; Nwosu et al., 2022; Trinidad et al., 2022).

The phenotypic spectrum of *SLC6A1*-related neurodevelopmental disorders (*SLC6A1*-NDD) includes intellectual disability (ID), autistic spectrum disorders (ASD), epilepsy, developmental delay, beginning from early infancy or after seizure onset, and neurological features such as hypotonia and movement disorders (Goodspeed et al., 1993; Carvill et al., 2015; Johannesen et al., 2018; Mattison et al., 2018; Cai et al., 2019; Devries et al., 2020; Goodspeed et al., 2020; Kahen et al., 2022). Indeed, the most consistent clinical features of the *SLC6A1*-NDD carriers are ID varying from mild to severe, behavioral and attention deficit hyperactivity disorders, and developmental delay that have been reported in almost all patients. Epilepsy is another hallmark of *SLC6A1*-deficiency related conditions, being reported in up to 82% of subjects in some studies (Kahen et al., 2022), featuring absences, myoclonic and atonic seizures as the most common seizure types. Additionally, roughly half of the subjects exhibit movement disorders, such as tremor, ataxia, or dystonic movements, tics; other common disturbances are sleep abnormalities, high pain tolerance, and ophthalmologic issues including myopia and strabismus. No clear genotype–phenotype correlations have emerged yet, as haploinsufficiency seems to be the underlying cause in both patients with truncating and missense variants. Inherited variants of *SLC6A1*, although less frequently, have been also described, however, no information has been reported yet on the variability of phenotypic features within families. Indeed, only one study reported on phenotypic heterogeneity primarily on epilepsy features in a couple of siblings (Poliquin et al., 2021).

Since most of the reported cases with a *SLC6A1* variant were relatively young patients, information on its course and long-term outcome are limited, thus studies on families might be useful to increase the knowledge on the phenotypic evolution and eventually to expand the phenotypic spectrum of *SLC6A1*-related conditions. In this study, we aim to describe the clinical phenotype in families with *SLC6A1* variants showing incomplete penetrance to investigate and outline possible intrafamilial variability.

## Methods

Patients with inherited variants of *SLC6A1* were collected through an international network of Epilepsy and Genetic Centers. Patient clinical, neurophysiological, and genetics data were collected and registered in a RedCap database (Harris et al., 2019) hosted at the Department of Epilepsy Genetics and Personalized Medicine at the Danish Epilepsy Center in Dianalund (Denmark). Probands were defined as the first person in a family to receive genetic counseling and/or testing for suspected hereditary risk; in some families two individuals (always siblings) were considered probands because both raised suspicion for a genetic disease. Carriers were defined as both the probands and the first-and second-degree relatives harboring a *SLC6A1* variant. Clinical data, mainly on epilepsy, neurodevelopment, behavioral features, and genotype were retrieved for this study. Seizures were classified according to the International League Against Epilepsy (ILAE) classification. Cognitive development, including intellectual and learning disabilities, was based on the assessment of the referring physicians. Variants are classified according to the ACMG criteria (Richards et al., 2015). Patients were included if they had a variant of unknown significance (VUS), a likely pathogenic or a pathogenic variant. One of the variants identified was found in a likely mosaic state in Genome Aggregation Database (gnomAD, https://gnomad.broadinstitute.org/).

The local ethical committees approved this study. All patients or parents/legal guardians in case of minors gave the consent to participate in the study.

## Results

We identified a total of 39 *SLC6A1*-deficiency carriers in 13 families. Two families (family D, family G) and subject II.1 from family I were already published in Johannesen et al. (2018), and family C was described in Kalvakuntla et al. (2023). Moreover, we included additional clinical information of three previously published cases: one (family A, IV.2) had only briefly been described previously in Goodspeed et al. (2020), two from family J (II.1 and II.2) were included in Halvorsen et al. (2016) only for the genetic data. Five probands from five families (II.1 from family B, III.1 from family E, II.1 from family F, II.1 from family H, and II.1 from family L) are going to be only briefly described in Stefanski et al. (submitted).

### Description of the families

#### Family A

This 12-year-old girl (IV.2) was born at term after an uneventful pregnancy, she was the second child of healthy unrelated parents. She crawled at 18 months and walked at 20 months. She first came to medical attention at 15 months for febrile seizures (FS). At the age of 3 years, she had her first afebrile generalized tonic clonic seizure. One year later, neurological evaluation showed impairment of both fine and gross motor skills, language delay, joint hypermobility, strabismus, hyperactive behavior, and difficulties to concentrate. The EEG at 5 years-old showed right-sided 3–5 Hz occipito-centro-temporal spikes/sharp waves followed by a slow wave, increasing during deep sleep.

At 10 years of age multifocal spikes/sharp-waves were observed. The MRI was normal. At the same age, she started to present atonic seizures, seizures with loss of contact associated with small myoclonic jerks, sleep-related focal seizures with variable semiology (left sided hypertonus, right sided clonia, eye deviation to the left, oro-alimentary automatisms, or eyes and head version to the right, loss of contact, and tachycardia). Valproate (VPA) was initiated, later shifted to levetiracetam (LEV) because of thrombocytopenia; under LEV, she presented very rare focal seizures out of sleep. At 11-year-old, genetic testing performed by whole exome sequencing, disclosed a maternally inherited likely pathogenic variant in *SLC6A1* c.283G > T, p. (Val95Phe). The mother (III.2) was completely asymptomatic, the maternal grandmother (II.2) with the same variant had some learning disabilities. The maternal uncle (III.1), also harboring the same variant, had some learning disabilities and was affected by alcohol addiction. The maternal great-uncle (II.5) was deceased, but his daughter (III.5) was harboring the same variant and presented ADHD and anxiety, while her daughter (IV.3) also harboring the same variant had febrile seizures plus, lasting until she was 9 years old, and presented some learning disabilities, being able to write and read but presenting with dysphasia.

#### Family B

This 15-year-old boy (II.1) with a moderate developmental delay, started to suffer from typical absence seizures at 36-month-old. The interictal EEG at the age of 11 years showed frequent generalized epileptiform discharges and generalized slow waves (<2.5 Hz) with anterior predominance, enhanced by NREM sleep. The brain MRI was normal. He was affected by anxiety, motor tics and tremor, unspecified sleep disturbances, and autistic features (mainly stereotypies). He achieved seizure freedom and his EEG normalized at the age of 13 years with an association of lamotrigine (LTG) and VPA.

A trio exome sequencing revealed a variant of unknown significance (VUS) in *SLC6A1* c.286C > G, p. (Pro96Ala) with paternal inheritance. The father (I.1) did not suffer from epilepsy but presented with a cognitive impairment with a borderline IQ.

#### Family C

Two adopted sisters, aged, respectively, 4 and 5 year-old (II.2 and II.1), were investigated for early developmental delay. Both presented the same VUS in *SLC6A1,* c.340 G > A; p. (Gly114Arg). Only few information are available about the medical history of the biological parents (I.1 and I.2): both presented some degree of mental illness and cognitive impairment, but none of them was genetically tested., The older sister (II.1) presented with developmental delay at the age of 2 years and absence seizures at the age of 4 years, followed by myoclonic seizures partially responsive to clobazam (CLB). The EEG before and after seizure onset was normal, displaying only occipital intermittent rhythmic delta activity (OIRDA). The neurological examination showed impaired coordination and extremely delayed and poorly articulated speech. MRI was unremarkable. She presented with ADHD features and had an aggressive behavior, worsened by VPA that was therefore discontinued.

The younger sister (II.2) showed developmental delay since the first year of life. The first absence seizures were noticed at the age of 2-year 8-month-old and atonic seizures at the age of 3 years. Both seizure types were partially responsive to CLB and VPA. The EEG showed occipital rhythmic delta activity. She was ataxic, exhibited a tremor (possibly related to VPA) and presented with self-aggressive and sensory seeking behavior, and she was diagnosed with autism spectrum disorder.

Both the sisters presented a speech delay and severe insomnia with fragmented sleep.

#### Family D

This 17-year-old man (II.2) presented with a mild developmental delay after birth. At 12 months, the patient started to present absence seizures and atonic seizures, partially controlled by VPA and ethosuximide (ESM), which were switched to lamotrigine (LTG) that was still not completely effective. After seizure onset, the ID was rated as moderate. The EEG showed 3 Hz generalized spike–waves (SW) discharges superimposed on a slow background activity. Neurological examination showed mild ataxia. Brain MRI highlighted a frontal widening of the lateral ventricles.

His 10-year-old sister (III.3) had absence and atonic seizures since childhood, controlled by ESM. The EEG showed slow background activity and generalized SW discharges. She presented with a mild ID. The mother (I.2) exhibited only mild learning disabilities. All three family members harbored the same likely pathogenic variant in *SLC6A1* c.695G > T, p. (Gly232Val).

#### Family E

This 9-year-old girl (III.1) was born from unrelated healthy parents. A developmental delay was diagnosed soon after birth, associated with unspecified sleep disturbances. At age 20 months, she started to suffer from myoclonic-atonic seizures, then during the course of the disease, she started to present also with absences and generalized tonic seizures. Seizures were refractory to antiepileptic treatments (lamotrigine, topiramate, and ketogenic diet). Ictal EEG showed slow background activity and frequent polyspike/spike–wave discharges associated with fluctuation of awareness and myoclonic jerks. She was unable to sit until the age of 22 months and did not walk until the age of 7 years. At this age, she presented with a severe ID, she was nonverbal with poor eye contact and autistic features. A pathogenic *SLC6A1* variant c.801delC, p. (Ile268Serfs*36) was found in the proband and also in the father (II.1) and in the grandfather (I.2). While the grandfather was completely asymptomatic, the father had a speech delay and learning disabilities.

#### Family F

A 39-year-old man (II.1) affected by severe ID, experienced his first absence seizures at age 5 months. Later on, he was diagnosed with Landau–Kleffner syndrome. He is nonverbal, his EEG showed 2.5–3 Hz spike–wave complexes predominant in the right hemisphere, especially in posterior temporal regions. Brain MRI was normal. The neurological examination showed pyramidal signs. He was positive for a likely pathogenic *SLC6A1,* c.889G > A; p (Gly297Arg). The same variant was also found in his 37-year-old sister (II.2), affected by a moderate ID and a speech impairment. She never had seizures and her EEG showed abundant slow waves in the anterior and central regions. They both inherited the variant from the 72-years-old (current age) father (I.1), affected by bipolar disorder.

#### Family G

This 18-years-old man (II.2), second child of unrelated parents, had a normal psychomotor development until the age of 2 years. At this age, he started to suffer from atypical absence seizures, associated in the follow-up with atonic and generalized tonic–clonic seizures, which were refractory to ASM. The EEG showed a background slowing and generalized SW discharges and multifocal spikes. After seizure onset, a cognitive regression was observed, and at the last follow-up he was diagnosed with moderate ID, and behavioral disorders (aggressiveness). Neurological examination showed hand tremor.

His 12-year-old brother (II.3) presented with a developmental delay since birth. At 15 months, he started to suffer from atypical absences, atonic seizures, and myoclonic-atonic seizures with similar EEG features as his older brother, not responsive to ASM. After seizure onset, the patient was diagnosed with a moderate ID with autistic features and occasional aggressive behavior. A mild hypotonia was found at neurological examination. The father (I.1) had learning disabilities and absence epilepsy since the age of 3 years, controlled with ESM. The therapy was discontinued at 17 years, and he is still seizure free.

All three subjects in this family harbored a likely pathogenic variant in *SLC6A1,* c.1024 G > A, p. (Val342Met).

#### Family H

This 11-year-old boy (II.1) started to present epileptic seizures characterized by eye blinking, change of facial expression and slight hypotonia at 23 months of age. The ictal EEG was characterized by generalized 3.5 Hz SW discharges with highest amplitude in the occipital regions and with alternating side of predominance. After VPA initiation, the EEG normalized, and the patient is currently seizure free. He was diagnosed with autism spectrum disorder and mild ID. Brain MRI was normal.

The likely pathogenic variant in *SLC6A1*, c.1070C > T, p. (Ala357Val), was identified in both the patient and the father (I.1), a 29 year-old man, affected by myoclonic-atonic seizures since the age of 4 years. He is seizure free with LEV, besides sporadic myoclonic jerks in the limbs. His EEG showed multifocal spikes, with right fronto-temporal predominance. He had a borderline IQ, and behavioral problems including obsessive–compulsive traits and lack of social inhibition.

#### Family I

This 21-year-old woman (III.1) was born after an uneventful pregnancy from a narcoleptic father and a dyslexic mother. Mild developmental delay was diagnosed soon after birth. At 7 years, she started to suffer from focal and generalized tonic–clonic seizures, which were controlled by LTG plus oxcarbazepine (OXC). The EEG showed prefrontal and frontal spikes. The MRI showed a malrotation of the left hippocampus. The pathogenic variant *SLC6A1* c.1084G > A, p.(Gly362Arg) was identified in the patient and in three family members: the sister (III.2), who went to special school because of some learning disabilities and presented with unspecified seizures, controlled by LTG, the mother (II.2), suffering from dyslexia, and a maternal uncle (II.I) with epilepsy onset at 25 years and unspecified seizure types.

#### Family J

**Figure 1:**
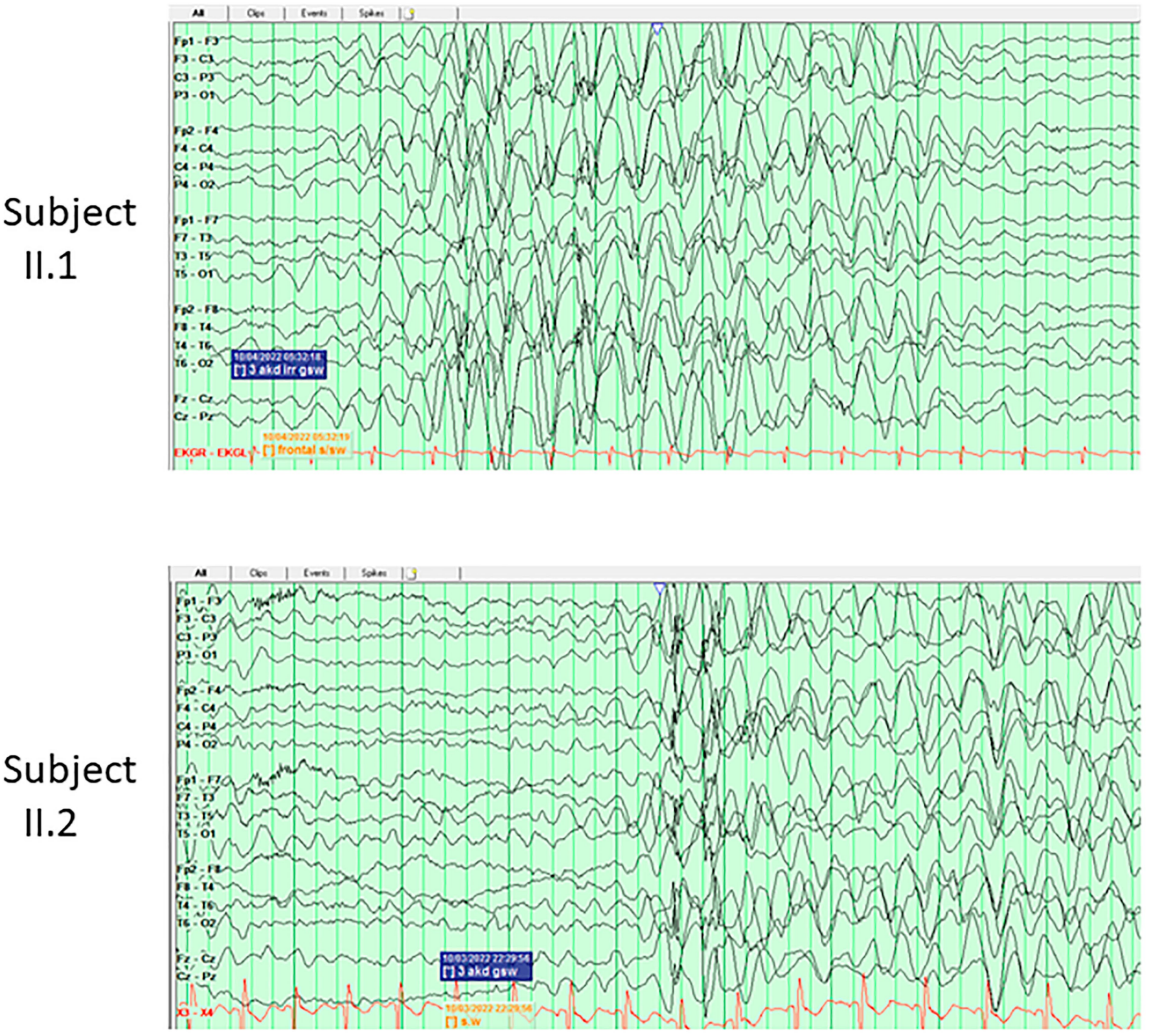
EEG tracings of the twins sisters (II.1 and II.2) of Family J, at the age of 3 years, showing in both subjects burst of hypersynchronous delta activities during wakefulness.

Two 3-year-old twin sisters (II.1 and II.2) were born from a healthy father (I.1) and a mother (I.2) who suffered from depression and anxiety. Both developed normally in the first months of life, then II.2 presented with a myoclonic-atonic seizure at 10 and half months, while II.1 started to show a slight developmental delay and suffered from her first myoclonic-atonic seizure at 12 months. In the follow-up, their development, although not formally tested, was reported as delayed. In both subjects, the EEGs at onset showed diffuse rhythmic delta slowing (Figure 1); while in the follow-up generalized bursts of SW during sleep were observed. Seizures were controlled by LTG, with the add-on of clorazepate in one of the two. Both brain MRIs had non-specific T2/FLAIR hyperintensities. II.1 had dysphagia and gastrointestinal disorders, while both presented ataxia and speech delay. They were diagnosed with a pathogenic *SLC6A1* variant, c.1084G > C, p.(Gly362Arg) inherited from the mosaic mother; the variant was present in approximately 18% of 62 reads in a buccal sample of the mother. Apart from the mentioned psychiatric comorbidities, the mother suffered from two isolated seizures provoked by Bupropion.

Two years later, a brother (II.3) was born, currently 1-year-old. He has the same *SLC6A1* variant, and he presented his first myoclonic-atonic seizure at 15 months. After seizure onset, he was diagnosed with a developmental delay associated with abnormal sensory processing, impairment in fine motor and visual motor skills. Topiramate was recently initiated. All three siblings display autistic features.

#### Family K

Two siblings, an 8-year-old girl (II.1) and a 6-year-old boy (II.2), were affected by the same likely pathogenic variant in *SLC6A1,* c.1328G > A, p. (Gly443Asp) inherited from germinal mosaicism in one of the parents. The girl started to walk at 14 months, the brother at 17 months. They both had language delay and ADHD. On Wechsler Intelligence Scale for Children (WISC), the sister had an IQ of 70, resulting from a Verbal Comprehension Index (VCI) of 75, Visual Spatial Index (VSI) of 77, Fluid Reasoning Index (FRI) of 74, Working Memory Index (WMI) of 88, and Processing Speed Index (PSI) of 71. She could recognize letters, but she could not read. Her brain MRI showed an enlargement of subarachnoid spaces and cortical sulci with fronto-temporal predominance.

The brother exhibited mild ID; neuropsychological testing showed the following scores: 50 in VCI, 85 in VSI, 80 in FRI, and 103 in WMI; IQ and PSI were uninterpretable. In addition, he displayed language and learning difficulties.

#### Family L

These two sisters of 56 and 51 years old (II.1 and II.2) presented with a family history of several relatives with intellectual disability and psychiatric disorders. They never had seizures, but both had mild ID. The older sister had autism spectrum disorder and borderline personality disorder, while the younger was affected by reactive attachment disorder. They both harbored the pathogenic variants *SLC6A1* c.1702C > T; p. (Gln568*) variant, found only 3 years ago.

#### Family M

An 8-years-old boy was born after an uneventful pregnancy from non-consanguineous parents. Since the first year of life, he showed a developmental delay. Head control was obtained at 7 months, rolled over at 11 months, stood at 22 months, and started walking at 24 months. His first words were at the age of 3 years, with the development of a partially intelligible speech only at 5.5-year-old. He is enrolled in a special needs school and has a mild ID (IQ 69), he presents an impairment in the understanding of complex tasks.

At 4- and 7-year-old, he presented two episodes of loss of consciousness, the cardiologic work-up resulted negative and was not investigated from a neurological point of view. Brain MRI showed nonspecific, diffuse white matter loss. He presented a plagiocephalic skull morphology.

Interestingly, all three elder siblings followed special education, mainly because of speech delay and learning problems. The father had dyslexia and followed special educations as well. In early childhood, various genetic analysis, including a trio exome study resulted negative. Later, in the course of the disease, a second trio exome, performed with special attention to possible inherited variants, revealed a paternally inherited pathogenic variant in *SLC6A1* c.1702C > T; p. (Gln568*). Segregation analysis in the siblings has not been performed yet.

### Summary of main clinical features

**Figure 2:**
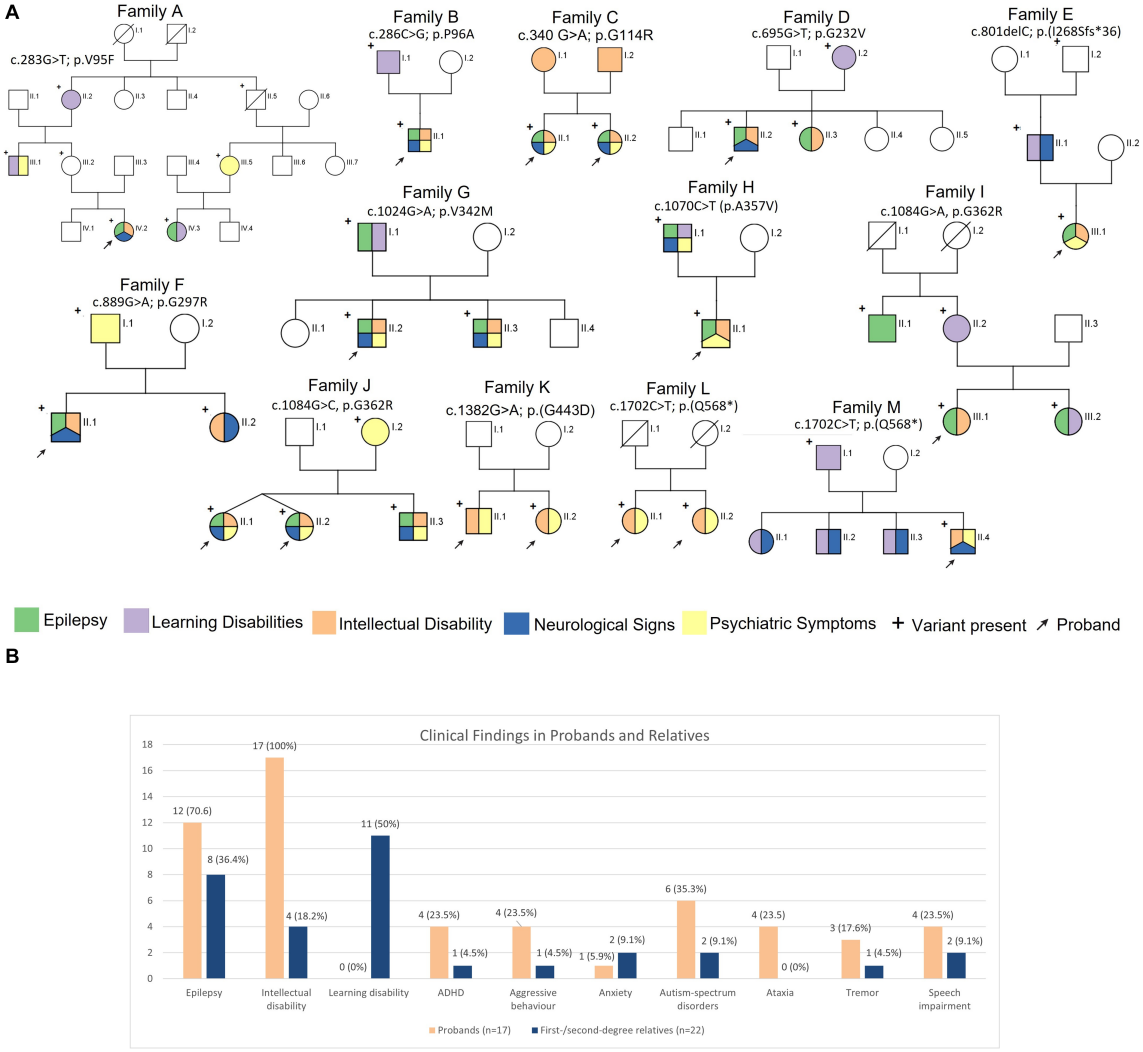
**(A)** Pedigrees of the 12 families reported in this study and main clinical features in the reported families. **(B)** Bar graph illustrating the prevalence of the neurological features in the probands and first/second degree relatives.

Main clinical features of the 13 families are illustrated in Figures 2A,B. Thirty-nine *SLC6A1*-deficiency cases, including 17 probands were investigated in this study. In the overall cohort, epilepsy was observed in 20/39 (51.3%) individuals. However, in the probands epilepsy occurred more frequently, being reported in 12/17 (70.6%), whereas it was observed in 8/22 (36.4%) of siblings or first-and second-degree relatives. Within a family the same epilepsy type, i.e., generalized or focal, was observed. All probands, but two (family A and family I) presented with generalized seizures as well as their siblings and first/second degree relatives. In families A and I, the probands and the other affected family members suffered from focal seizures.

In those patients where the information on seizure types were available, the most common type was absence seizures reported in 12/20 (60%) individuals, followed by myoclonic-atonic seizures in 7/20 (35%), atonic in 6/20 (30%), generalized tonic–clonic seizures in 4/20 (20%), focal seizures in 2/20 (10%), and febrile seizures plus in 1/20 (5%). Febrile seizures were described in 3/20 (15%). Ten patients (50%) presented multiple seizure types, including a combination of generalized and focal in two of them (IV.2 family A, III.1 family I).

Overall ID was reported in 21/39 (53.8%) individuals, including the 17 probands; only 4 (12.8%) siblings or first/second-degree relatives presented with some degree of ID. Learning disabilities or borderline IQ were diagnosed in 11/39 patients (28.2%), all of them were relatives of a proband affected by ID, diagnosed as part of a segregation study.

Twenty out of 39 individuals (51.3%) presented with psychiatric symptoms or behavioral disorders, including 14/17 (82.4%) probands. Out of the 20 patients with psychiatric symptoms, eight (8/20, 40%) were diagnosed with ASD, seven also being affected by epilepsy. Other less common symptoms were anxiety (3/20, 15%), aggressive behavior (5/20, 25%), and ADHD (5/20, 25%). It is notable that ADHD was found in two siblings that besides ADHD, presented only intellectual disability. Depression, borderline personality, post-traumatic stress disorders, reactive attachment disorder, obsessive–compulsive traits, sensory seeking disorder, and bipolar disorder were reported in single cases.

Neurological disturbances were seen in 15/39 (38.5%) cases including 10 probands (58.8%). Most common neurological disturbances were tremor and ataxia (both seen in 4/15, 26.7%) and speech difficulties (in 6/15, 40%). The neurological symptoms were reported in 5/22 (22.7%) of the relatives, and consisted of speech impairment in 2/5, hypotonia, impairment of fine motor skills, and intentional and postural tremor in single cases.

### Genetics

**Figure 3:**
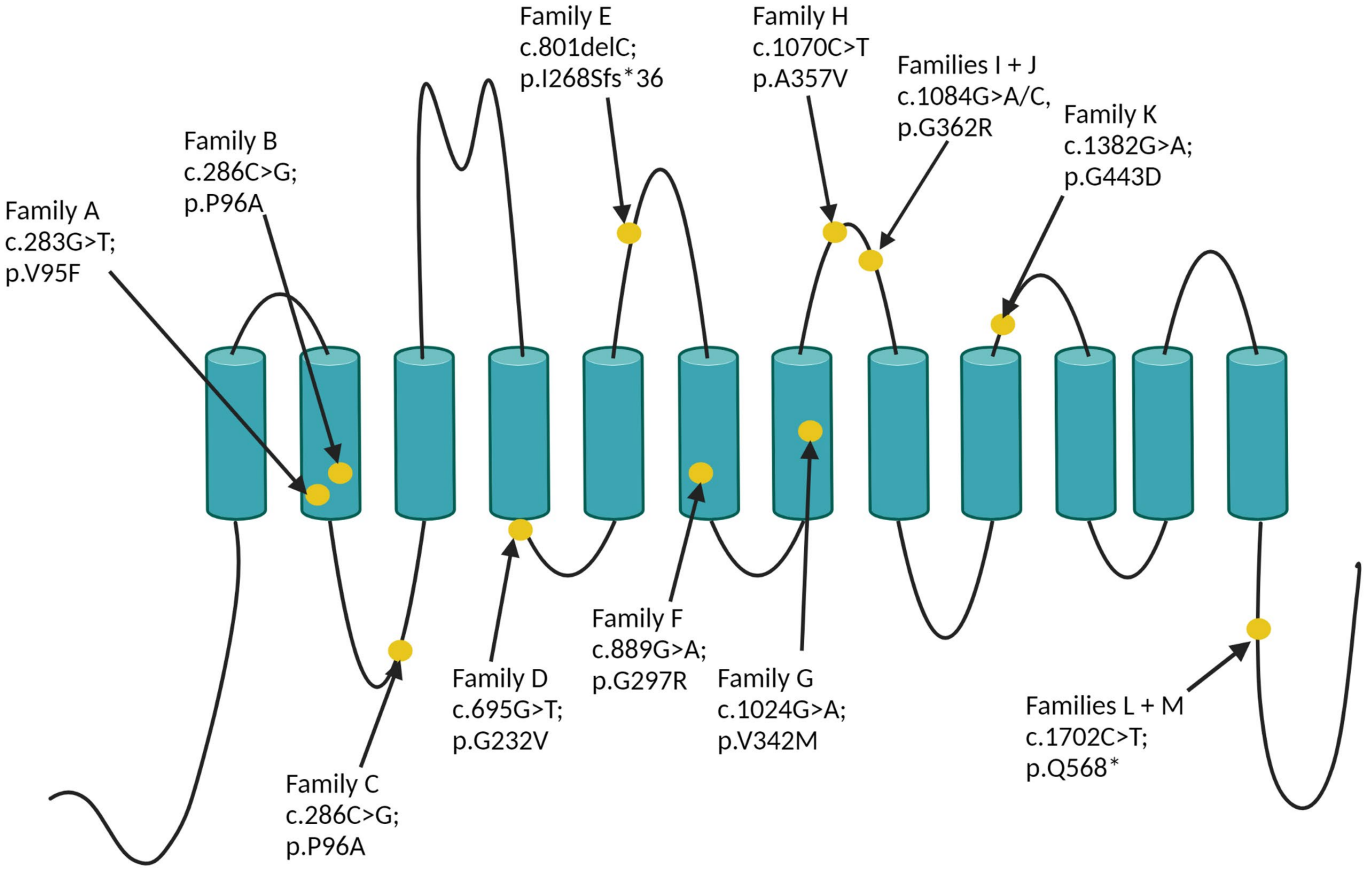
The 12 SLC6A1-variants displayed on GAT-1.

The identified *SLC6A1* variants are reported in Table 1 and displayed in Figure 3. Per definition all variants were inherited, and family segregation was performed in older generations to attempt confirmation of the origin. One variant was a frameshift (family E), one was a nonsense, and the remaining were missense variants. According to the ACMG criteria two variants were classified as VUS (III), six as likely pathogenic (IV) and three as pathogenic (Table 1). The following variants were novel: c.286C > G; p.Pro96Ala, c.340G > A; p.Gly114Arg, c.801delC; p.(Ile268Serfs*36), c.1024G > A; p.Val342Met, and c.1702C > T; p.(Gln568*). Four variants were recurrent seen in >2 families: c.889G > A; p.Gly297Arg, c.1024 G > A; p.(Val342Met), c.1070C > T; (p.Ala357Val), and c.1084G > A; p.(Gly362Arg), of which the latter was also recurrent within this study. Furthermore, the nonsense variant c.1702C > T; p.(Gln568*) was seen in two families within this study (families L and M). All variants were absent from GnomAD, except for the recurrent c.1024 G > A, p.(Val342Met) variant, which was observed once (1/251402) in a likely mosaic state.

**Table 1:** SLC6A1 variants and frequency of the main clinical features in each family.

Family	Variant NM_003042.4/N P_003033.3	CADD score	Classification (III–V)	SIFT (Score)	PolyPhen (Score)	Mutation Taster	Epilepsy	Intellectual disability	Psychiatric symptoms	Learning disabilities	Neurological signs
A	c.283G>T; p.(V95F)	24.4	IV (PS4, PM2, PP1, PP3)	Deleterious (0)	Possible damaging (0.649)	Benign	2/7	1/7	2/7	3/7	1/7
B	c.286C>G; p.(P96A)	24.8	III (PM2, PP3)	Deleterious (0)	Possible damaging	Deleterious	1/2	1/2	1/2	1/2	1/2
C	c.340G > A; p.(G114R)	27.9	III (PM2, PP3)	Deleterious (0.03)	Probably damaging	Deleterious	2/2	2/2	2/2	0/2	2/2
D	c.695G>T; p.(G232V)	28.6	IV (PS4, PM2, PP1, PP3)	Deleterious (0)	Probably damaging (1)	Deleterious	2/3	2/3	0/3	1/3	1/3
E	c.801delC; p.(G297R)	-	V (PVS1, PM2, PP3)	NA	NA	-	1/3	1/3	1/3	1/3	1/3
F	c.889G>A; p.(G297R)	25.5	IV (PS4, PM2, PP1, PP3)	Deleterious (0.02)	Benign (0.373)	Deleterious	1/3	2/3	1/3	0/3	2/3
G	c.1024G>A; p.(V342M)	28.5	IV (PS4, PM2, PP1, PP3)	Deleterious (0)	Probably damaging (0.967)	Deleterious	3/3	2/3	2/3	1/3	2/3
H	c.1070C>T; p.(A357V)	31	IV (PS4, PM2, PP1, PP3)	Deleterious (0.02)	Probably damaging (0.91)	Deleterious	2/2	1/2	2/2	1/2	1/2
I	c.1084G>A, p.(G362R)	24.9	V (PS4, PM1, PM2, PP1, PP3)	Deleterious (0)	Possible damaging (0.864)	Deleterious	3/4	1/4	0/4	2/4	0/4
J	c.1084G>A, p.(G362R)	24.7	V (PS4, PM1, PM2, PP1, PP3)	Deleterious (0)	Possible damaging (0.864)	Deleterious	3/4	3/4	4/4	0/4	3/4
K	c.1328G>A; p.(G443D)	27.1	IV (PS4, PM2, PP3)	Deleterious (0)	Probably damaging (1)	Deleterious	0/2	2/2	2/2	0/2	0/2
L	c.1702C>T; p.(Q568*)	41	V (PVS1, PS4, PM2)	-	-	-	0/2	2/2	2/2	0/2	0/2
M	c.1702C>T; p.(Q568*)	41	V (PVS1, PS4, PM2)	-	-	-	0/2	1/2	1/2	1/2	1/2

The recurrent c.1084G > A, p.(Gly362Arg) variant is located in a mutational hotspot with high pathogenic missense burden within the *SLC6A6-14* gene family, and the c.1070C > T (p.Ala357Val) variant is located nearby.^1^ The CADD score for the missense/nonsense variants ranges between 24.4 and 41.^2^

Ten out of 12 variants were associated with epilepsy in this study. The probands and the siblings reported in family K, L and M did not suffer from epilepsy. Family K was diagnosed with a missense variant [c.1328G > A; p.(Gly443Asp), VUS] and family L and M with a truncating variant [c.1702C > T; p.(Gln568*)]. No genotype–phenotype associations were identified.

## Discussion

Here we present a cohort of 13 families and 39 *SLC6A1*-carriers in total, including 17 probands. In this cohort, the probands exhibited as main common clinical features ID, psychiatric/behavioral disorders and epilepsy in 100, 82.4, and 71% of individual, respectively. These findings replicate previous cohort studies on the phenotypic expression of *SLC6A1* deficiency (Goodspeed et al., 1993; Carvill et al., 2015; Johannesen et al., 2018; Kahen et al., 2022).

Most of the probands (71%) in our study presented with epilepsies with generalized seizures, such absences (typical and atypical) and myoclonic-atonic seizures, as previously reported. Focal seizures were reported only in two probands, one of them presenting a developmental encephalopathy with multifocal EEG epileptic abnormalities and a combination of focal (possibly frontal) and generalized seizures, whereas in the other individual the seizure semiology was not specified. Indeed, focal seizures in *SLC6A1*-NDD have been rarely reported. Only Johannesen et al. (2018) described focal seizures (possibly involving the temporal or central regions) in three subjects, whose family history was negative or unknown.

In siblings and first/second degree-relatives, epilepsy occurred more rarely, being diagnosed only in 36.4% of individuals. In subjects in whom the information was available, we found that within the same family there was a consistent overlap among seizure types, with most of patients and relatives presenting with generalized seizures, in particular absences. Finally, susceptibility to fever was reported in two individuals in family A, presenting with febrile or febrile seizures plus. EEG findings confirmed the clinical diagnosis of generalized or focal epilepsy, showing generalized spike–wave discharges or focal abnormalities, respectively. Interestingly, OIRDA was observed in both probands in family C, suffering from absence and myoclonic seizures, replicating a previous EEG observation by Poliquin et al. (2021). In addition, the probands in family F and H, displayed a peculiar EEG pattern characterized by diffuse spike-and wave discharges with posterior predominance, an EEG feature previously reported in a proportion of individuals by Johannesen et al. (2018), possibly suggesting this EEG feature as a possible EEG biomarker for *SLC6A1*-NDD. Unfortunately, EEG data were lacking in most of siblings and on first-second degree relatives, so this latter finding cannot be further corroborated by our study.

The finding of ID, from mild to severe, in 100% of probands (with or without epilepsy), confirms that impairment of cognition of variable degree is one of the cardinal features of *SLC6A1*-NDD (Johannesen et al., 2018). However, the incidence of ID decreases sharply in the relatives, being observed only in four out of 22 (18.2%) siblings and first/second-degree relatives. In addition, in all these subjects, ID was reported to be mild to moderate and never severe. Interestingly, learning disabilities or borderline IQ were diagnosed in 11/39 individuals (28.2%), all of them were siblings or first/second-degree relatives of a proband affected by severe–moderate ID. As for ID, also ASD and autism were more frequent in probands, being diagnosed in 35.3% of them (6/17), whereas only 9.1% (2/22) of siblings or first/second-degree relatives presented with these disorders.

Thus, the analysis of the intrafamilial phenotypic expression showed that first-or second-degree relatives presented with an incomplete, less complex and less severe clinical picture featuring only epilepsy in three families (G, H, and I) and otherwise mainly mild intellectual and/or learning disabilities. These less severe phenotypic expressions contrasted with the main clinical findings observed in the probands, which included ID from moderate to severe, generalized epileptic seizures, in some patients refractory to treatment, behavioral, and psychiatric disorders. A striking example is represented by family E where the grandfather is unaffected, the father presents a learning disability, and the proband has a refractory epilepsy and severe ID.

Several factors may explain the observed intrafamilial variability. Firstly, it is well-known that genetic diseases are often described initially as a homogenous, often severe disorder. Indeed, in the case of *SLC6A1*, the initial core phenotype was that of myoclonic-atonic epilepsy associated with language disorders and various degrees of ID (Carvill et al., 2015; Johannesen et al., 2018), while variability in phenotypic expression in *SLC6A1* families has been poorly explored, and very few familial cases have been reported, usually included in larger cohorts (Carvill et al., 2015; Johannesen et al., 2018; Goodspeed et al., 2020). As more patients are discovered the phenotypic spectrum usually expands, often including milder forms. Thus, the phenotypic variability described in this study may simply be a further widening of the phenotypic spectrum. As first/second degree relatives were less severely affected, typically these individuals would not necessarily be clinically investigated, and even more likely would not be submitted to genetic testing. Thus, they will go undiagnosed unless a family member develops a more severe phenotype prompting genetic testing. Additionally, some of the families presented here were detected as part of genetic trio WES studies, which in general is the preferred analysis when suspecting a genetic disorder. However, the risk of trio analysis is that with lack of sufficient information of more subtle symptoms in parents or relatives, inherited variants may be overlooked during the data analysis, particularly when the search is for *de novo* variants, and thus the patient may be classified as genetically undiagnosed.

Secondly, we acknowledge that the *SLC6A1* variant found in these families might not be the sole cause of disease in the most affected family members. It is well known that other genetic factors, as well as environmental factors may influence the phenotypic outcome. In a recent *SCN1A* study on a cohort of patients with Dravet syndrome (Martins Custodio et al., 2023) it has been demonstrated that *SCN1A* pathogenic variants did not necessarily acted alone in producing the final phenotype. Indeed, DS-causing pathogenic variants might need to act against a largely compromised genomic background (such as, for instance, a lower polygenic risk scores—PRS—for intelligence) to result in the complete Dravet syndrome phenotype.

If the above findings for *SCN1A* can be extrapolated to other genes such as *SLC6A1*, that would be an additional explanation of the observed phenotypic variability.

*SLC6A1*-NDD are thought to be caused by haploinsufficiency of the GAT-1 gene product. However, even though a plethora of missense variants have been reported in *SLC6A1*-NDD, it is challenging to interpret the pathogenicity of missense variants in *SLC6A1*. Thus, reclassification of variants from VUS to likely pathogenic or pathogenic variants largely depends on *de novo* inheritance, a previous pathogenic report of the same variant or functional testing (Richards et al., 2015). As functional testing of missense variants is not readily available in clinical settings, the pathogenicity of many of these variants will remain unknown until further studies are conducted.

Our study has some limitations, besides the incomplete knowledge of the pathogenicity of some of the variants reported here. In particular, data from first-and more often second-degree relatives were incomplete in some families, thus limiting the possibility to provide a more precise phenotypic description. In addition, since this is a multicenter study, the assessment of some clinical features was not homogeneous, in particular regarding ID and learning disabilities, that in some individuals (particularly in first/second-degree relatives) was based on clinical evaluation and not on formal neuropsychological testing.

In conclusion, this study is the first to systematically show a variable intrafamilial phenotypic expression of *SLC6A1*-NDD, however further investigations are necessary to confirm and eventually further expand the phenotypic spectrum of this disorders. We speculate that a precise diagnosis for *SLC6A1*-NDD, is often made in subjects with overt clinical symptoms, thus overlooking subjects with a milder clinical presentation, even belonging to a family where symptomatic carriers might have been diagnosed. Pre-test genetic counseling and a detailed collection of family history may help to avoid filtering out causative variants because also found in apparently healthy first−/second-degree relatives.

Like in other developmental epileptic encephalopathies, a proportion of individuals with mild *SLC6A1*-NDD might be missed, in particular those of older age, where genetic testing is often not considered. Additionally, the attention to genetic causes of ID and behavioral disorders has increased over the years, thus in the past these disorders, particularly when mild, might have passed unnoticed. Future research should aim to collect additional information on familial variability and to further investigate the pathogenicity of *SLC6A1* variants to obtain a more comprehensive view of the phenotypic heterogeneity of *SLC6A1*-NDD. This, besides expanding the *SLC6A1*-NDD phenotypic spectrum, might allow the identification of genotype–phenotype correlations and provide a genetic basis for more accurate genetic counseling.

## Data availability statement

The raw data supporting the conclusions of this article will be made available by the authors, without undue reservation.

## Ethics statement

The studies involving human participants were reviewed and approved by Local ethical Committees. Written informed consent to participate in this study was provided by the participants’ legal guardian/next of kin. Written informed consent was obtained from the individual(s), and minor(s)’ legal guardian/next of kin, for the publication of any potentially identifiable images or data included in this article.

## Author contributions

BK, KJ, CF, RM, and GR reviewed the literature, performed clinical data collection, and drafted the manuscript. MW, AA-S, TL, PM, LL, BJ, MB, AK-M, AV, GV-L, HA-M, KG, MS, and TB contributed to collecting clinical data. GR, KJ, and RM conceived the idea of the present paper, and critically reviewed and finally approved the manuscript. All authors contributed to the article and approved the submitted version.

## Conflict of interest

KG has consulted for Taysha Gene Therapy, Jaguar Gene Therapy, Astellas Gene Therapy, and all Stripes for unrelated work. She also serves as co-chair of the Clinical Advisory Board (unpaid) for the non-profit COMBINEDBrain, which involves collaborations on *SLC6A1*-NDD.

The remaining authors declare that the research was conducted in the absence of any commercial or financial relationships that could be construed as a potential conflict of interest.

## Publisher’s note

All claims expressed in this article are solely those of the authors and do not necessarily represent those of their affiliated organizations, or those of the publisher, the editors and the reviewers. Any product that may be evaluated in this article, or claim that may be made by its manufacturer, is not guaranteed or endorsed by the publisher.

## Footnotes

^1^ https://per.broadinstitute.org/

^2^ https://cadd.gs.washington.edu/snv
