# Corrigendum: Intrafamilial variability in *SLC6A1*-related neurodevelopmental disorders

**DOI:** 10.3389/fnins.2023.1270299

**Published:** 2023-08-11

**Authors:** Benedetta Kassabian, Christina Dühring Fenger, Marjolaine Willems, Angel Aledo-Serrano, Tarja Linnankivi, Pamela Pojomovsky McDonnell, Laina Lusk, Birgit Susanne Jepsen, Michael Bayat, Anja A. Kattentidt-Mouravieva, Anna Abulí Vidal, Gabriel Valero-Lopez, Helena Alarcon-Martinez, Kimberly Goodspeed, Marjon van Slegtenhorst, Tahsin Stefan Barakat, Rikke S. Møller, Katrine M. Johannesen, Guido Rubboli

**Affiliations:** ^1^Department of Epilepsy Genetics and Precision Medicine, Danish Epilepsy Center, Member of the European Reference Network EpiCARE, Dianalund, Denmark; ^2^Neurology Unit, Department of Neuroscience, University of Padua, Padua, Italy; ^3^Amplexa Genetics, Odense, Denmark; ^4^Département Génétique Médicale, Maladies Rares et Médecine Personnalisée, Hôpital Arnaud de Villeneuve, CHU de Montpellier Institute for Neurosciences of Montpellier, Univ Montpellier, INSERM, Montpellier, France; ^5^Epilepsy and Neurogenetics Program—Vithas Madrid La Milagrosa University Hospital, Vithas Hospital Group, Madrid, Spain; ^6^Department of Pediatric Neurology, New Children's Hospital and Pediatric Research Center, Epilepsia Helsinki, Helsinki University Hospital and University of Helsinki, Helsinki, Finland; ^7^Division of Neurology, Children's Hospital of Philadelphia, Philadelphia, PA, United States; ^8^Department of Neurology, University of Pennsylvania Perelman School of Medicine, Philadelphia, PA, United States; ^9^Epilepsy Neurogenetics Initiative, Division of Neurology, Children's Hospital of Philadelphia, Philadelphia, PA, United States; ^10^Pediatric Department, Danish Epilepsy Center, Dianalund, Denmark; ^11^Department of Neurology and Center for Rare Diseases, Aarhus University Hospital, Aarhus, Denmark; ^12^Stichting Zuidwester, Middelharnis, Netherlands; ^13^Department of Clinical and Molecular Genetics, University Hospital Vall d'Hebron and Medicine Genetics Group Vall d'Hebron Research Institute (VHIR), Barcelona, Spain; ^14^Neurology Department, Virgen de la Arrixaca University Hospital, Murcia, Spain; ^15^Department of Pediatric Neurology, Virgen de la Arrixaca University Hospital, Murcia, Spain; ^16^Department of Pediatrics, Division of Neurology, University of Texas Southwestern Medical Center, Dallas, TX, United States; ^17^Department of Neurology, University of Texas Southwestern Medical Center, Dallas, TX, United States; ^18^Department of Clinical Genetics, Erasmus MC University Medical Center, Rotterdam, Netherlands; ^19^Discovery Unit, Department of Clinical Genetics, Erasmus MC University Medical Center, Rotterdam, Netherlands; ^20^ENCORE Expertise Center for Neurodevelopmental Disorders, Erasmus MC University Medical Center, Rotterdam, Netherlands; ^21^Institute of Regional Health Research, University of Southern Denmark, Odense, Denmark; ^22^Department of Genetics, University Hospital of Copenhagen, Rigshospitalet, Copenhagen, Denmark; ^23^Department of Clinical Medicine, Faculty of Health and Medical Sciences, University of Copenhagen, Copenhagen, Denmark

**Keywords:** SLC6A1, intrafamilial variability, epilepsy, neurodevelopmental disorders, intellectual disability

In the published article, there was an error in affiliation 12. Instead of “Genetic Department, Stichting Zuidwester, Middelharnis, Netherlands”, it should be “Stichting Zuidwester, Middelharnis, Netherlands.”

In the published article, an author name was incorrectly written as Anja Kattentidt. The correct spelling is Anja A. Kattentidt-Mouravieva.

The initials in author contributions should not be AK but AK-M.

The authors apologize for this error and state that this does not change the scientific conclusions of the article in any way. The original article has been updated.

